# Modulating Fish Gelatin Gelling Properties Through Furcellaran Addition: A Structural and Physicochemical Analysis

**DOI:** 10.3390/gels11060381

**Published:** 2025-05-23

**Authors:** Tanyamon Petcharat, Manat Chaijan, Sylvia Indriani, Jaksuma Pongsetkul, Supatra Karnjanapratum, Sitthipong Nalinanon

**Affiliations:** 1 School of Food Industry, King Mongkut’s Institute of Technology Ladkrabang, Ladkrabang, Bangkok 10520, Thailand; tanyamon.pe@wu.ac.th; 2 Professional Culinary Arts Program, School of Management, Walailak University, Nakhon Si Thammarat 80161, Thailand; 3 Food Technology and Innovation Research Centre of Excellence, School of Agricultural Technology and Food Industry, Walailak University, Thasala, Nakhon Si Thammarat 80161, Thailand; cmanat@wu.ac.th; 4 School of Animal Technology and Innovation, Institute of Agricultural Technology, Suranaree University of Technology, Nakhon Ratchasima 30000, Thailand; indrianisylvia@gmail.com (S.I.); jaksuma@sut.ac.th (J.P.); 5 Division of Marine Product Technology, Faculty of Agro-Industry, Chiang Mai University, Chiang Mai 50100, Thailand; supatra.ka@cmu.ac.th

**Keywords:** furcellaran, fish gelatin, gelatin substitute, gelling property, microstructure

## Abstract

Fish gelatin (FG) is a promising alternative to mammalian gelatin but is limited by poor gelling ability, low gel strength, and inability to set at room temperature. This study evaluated the effects of furcellaran (FUR), a gelling agent, on the structural and physicochemical properties of FG gels at different substitution levels (25–100%). The addition of 25% FUR improved gel strength and hardness. However, higher FUR levels (>50%) led to reduced springiness and increased syneresis. Intermolecular force measurements revealed that ionic and hydrogen bonds were crucial in the FG/FUR gel system, with higher levels of FUR promoting stronger ionic and hydrogen bonding. Color changes were observed with decreased L* and increased b* and ∆E* values as FUR levels rose. Gelling and melting points also increased proportionally with FUR content. Microstructural analysis showed denser gel networks with smaller gaps upon FUR incorporation. SAXS analysis confirmed enhanced structural conformation with higher FUR levels. An appropriate level of FUR added (25%) could therefore improve gelling properties via increasing gel strength and gelling temperature without negative effects on springiness and syneresis of resulting gel.

## 1. Introduction

The lack of collagen as part of human organisms and tissues contributes to one of the main causes leading to the aging of tissues, the deterioration of organ function, and the onset of chronic illnesses [1]. The supplementation of collagen is a significant method for minimizing the effects of aging while encouraging optimal wellness [2,3,4]. In contrast to collagen peptides, gelatin exhibits diverse functional properties, including gelling, foaming, and emulsifying [5]. Gelatin can produce many collagen-based nutrition foods, such as jellies, gummies, and pastries. Consequently, gelatin has the potential to emerge as a focal point of research as a prospective carrier for collagen normalization additives.

The global demand for gelatin has been rising in the food-related and medical sectors. Mammalian gelatins, specifically from bovine and porcine sources, are extensively utilized. Consumer restrictions and skepticism, triggered by sociocultural factors, such as religion and medical conditions, played an important part in the emergence of alternative gelatin [6]. Fish gelatin has received interest from kosher and halal markets, primarily derived from undesirable byproducts of the fishing industry, including skin and scales [7,8]. However, fish gelatin exhibits poor gelling properties, characterized by low gel strength and low gelling temperature (could not set gel at room temperature), which inhibits its applications [9]. To overcome this limitation, various physical methods, including ultrasound, irradiation, and electrolytes [10], as well as cross-linking enzymes like microbial transglutaminase [11,12,13], and natural protein cross-linkers, specifically phenolic compounds extracted from several plants, have been applied to enhance the strength of fish gelatin gels [14,15,16]. Additionally, several hydrocolloids, such as agar [17], κ-carrageenan [18,19,20], pectin [21,22], and gellan [23,24,25,26,27] have been documented to enhance the gelling characteristics of fish gelatin.

Furcellaran is an anionic polysaccharide, which could provide the negative charge, and is derived from red algae known as *Furcellaria lumbricalis*. It is a linear biopolymer made out of components constituting a segment of 3-linked β-D-galactopyranose with a sulfate group at the carbon 4 position and 4-linked 3,6-anhydro-α-D-galactopyranose [28]. Furcellaran is a chemical structure associated with *κ*-carrageenan, but furcellaran possesses a lesser degree of sulfation. Anionic polysaccharide furcellaran is currently recognized as a highly promising biopolymer for producing biodegradable films [29]. Additionally, the incorporation of 6% furcellaran could enhance the gelling qualities of surimi gel, resulting in increased gel strength without negatively impacting the sensory characteristics of the final product [30]. Despite its promising functional characteristics, the application of furcellaran in gelatin-based hydrogels, particularly those derived from fish sources, has not been studied. Compared to previous work on surimi gels (myofibrillar protein systems), the effect of furcellaran on fish gelatin gels (hydrogels) remains unexplored. Therefore, this study aims to evaluate the influence of furcellaran on the gelling properties of fish gelatin gels. The findings are expected to contribute to the development of improved marine-based hydrogel systems and provide new insights into the use of furcellaran in fish gelatin formulations.

## 2. Results and Discussion

### 2.1. Textural Properties of FG Gels as Influenced by FUR Addition

#### 2.1.1. Gel Strength

**Figure 1:**
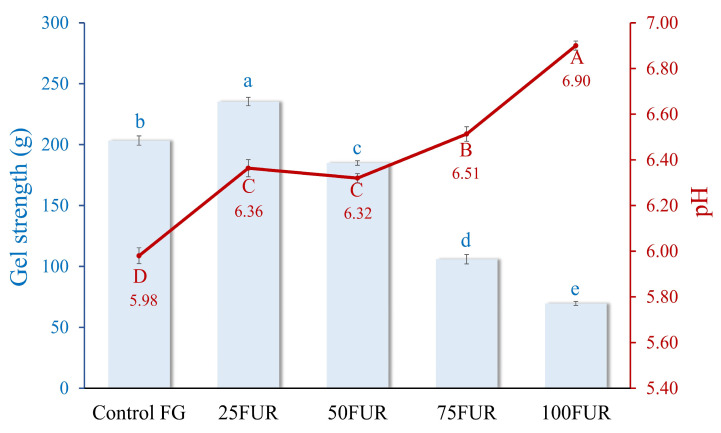
Gel strength and pH of fish gelatin (FG) as affected by the addition of furcellaran (FUR) at different levels. Bars and the line represent gel strength and pH, respectively. The bars and line are presented as the standard deviation (*n* = 10). Lowercase or uppercase letters on the bar and line indicate significant differences (*p* < 0.05).

Gel strength is critical for determining its suitability in various food applications. The addition of furcellaran, a polysaccharide derived from red algae, at different levels, significantly influences gel strength, as illustrated in Figure 1. When the content of FUR was increased up to 25%, the gel strength of FG/FUR blended gels was enhanced (*p* < 0.05). The control FG gel showed a lower gel strength (203.34 g) in comparison to the 25FUR gel (*p* < 0.05). An enhancement in the gel strength of FG incorporated with FUR might be attributed to the interactions between FG and FUR, leading to a robust network. FUR has the potential for generating ‘new heterolytic junction zone networks’ with the molecules of gelatin. As shown in Figure 1, the pH values of the resulting gels were in the range between 5.5 to 7. This indicated that the FG should be delivering positive charges, as the pH values of the FG gel containing FUR remained below the isoelectric point (pI) of the FG (the pI of FG was pH ~ 8.8) [21]. Therefore, positively charged residues of proteins can interact with negatively charged sulfate group domains in furcellaran through ionic interaction, which was illustrated by the greater gel strength [30,31]. Additionally, the interactional effects that occur between two biopolymers (FG and FUR), which include hydrogen bonding and hydrophobic interactions, might also be responsible for the strengthening of mixed gels.

A similar phenomenon also occurred in gelatin containing κ-carrageenan; the rise in gel strength of gelatin-based composite gel was reported when an appropriate amount of κ-carrageenan was added. Gel networks with superior gelation properties could be formed by clustering gelatin and the molecules of κ-carrageenan within helices through hydrogen bonding and electrostatic interactions [18]. As a result, a higher level of FUR (>50%) led to a decline in gel strength. FUR might promote excessive cross-linking between fish gelatin molecules, which can lead to a denser, less flexible network. Chen, et al. [32] found that while cross-linking enhances gel strength, excessive cross-linking can lead to a rigid network that may compromise the gel’s flexibility and overall strength. Instead of forming a uniform and ordered structure, the gel may become overly rigid, preventing the formation of the fine, ordered network required for optimal gel strength. This imbalance in cross-linking could reduce the ability of the gel to withstand deformation, thus lowering its gel strength. Moreover, high levels of furcellaran might cause phase separation between the hydrocolloid and the gelatin proteins. This separation can lead to heterogeneous gel structures, weakening the overall gel matrix and reducing the cohesion of the network, further contributing to a decrease in gel strength [19,33]. Consequently, the incorporation of furcellaran under optimal conditions effectively strengthens the gel strength of fish gelatin.

#### 2.1.2. Texture Profile Analysis (TPA)

TPA can imitate alterations in the physical characteristics of hydrocolloids during mastication. The parameters assessed, including hardness, springiness, cohesiveness, gumminess, and chewiness of FG affected by different concentrations of FUR, are shown in Table 1. The hardness of the resultant gel escalated with the increment of FUR concentration up to 25%, in which the gel containing 25% FUR had the highest hardness when compared to all samples (*p* < 0.05). The interaction between FG (positively charged chains) and FUR (negatively charged sulfate ester groups) might be contributing to the increase in hardness of the FG/FUR gels. However, with the addition of FUR higher than 50%, the resulting mixed gel showed a lower hardness (*p* < 0.05). The changes in hardness when FUR levels increased were consistent with the changes in gel strength of FG/FUR mixed gels, as shown in Figure 1. Springiness, also known as “elasticity,” is the rate at which a deformed material returns to its undeformed state after removing the deforming force. It refers to a subjective perception of “rubberiness” during mastication [34,35].

It was remarked that the control sample and the mixed gel added with 25% of FUR had no significant difference in springiness (*p* > 0.05). Comparatively, the gel samples with a high level of FUR (>50%) showed a dramatic decrease in springiness. Adding FUR rendered the FG gel more brittle, resulting in decreased springiness. In general, flexibility and elasticity have been claimed as the necessary characteristics of gelatin when applied to foods [36,37]. Incorporating FUR at high concentrations enhanced rigidity or brittleness, adversely impacting the textural characteristics of FG/FUR mixed gels, evident by a reduction in springiness. However, when compared with other hydrocolloids (gellan, pectin, and κ-carrageenan) in previous studies, FUR had a lower adverse effect on springiness than gellan, pectin, and κ-carrageenan [19,21,27]. Furthermore, the incorporation of FUR drastically lowered the cohesiveness of FG gel (*p* < 0.05), while trends in gumminess and chewiness of FG/FUR mixed gel are similar to the trend in hardness. Previous studies by Kaewruang, et al. [38], Wangtueai, et al. [39], Liu, et al. [40] have reported a correlation between the hardness of gelatin and its other textural properties, particularly gumminess and chewiness. These parameters are closely linked because increased hardness often contributes to a denser gel matrix, which in turn enhances the resistance to deformation (gumminess) and the energy required to masticate the gel (chewiness). Overall, texture profile data suggested that FUR, at low concentrations, could enhance the gel strength and overall textural characteristics. Specifically, 25% FUR increases hardness, gumminess, and chewiness without adverse effects on springiness. This indicated that FUR, at low concentrations, acts as a reinforcing agent for the gel network structure by enhancing the interaction between FG and FUR molecules.

### 2.2. Intermolecular Forces of FG Gels as Influenced by FUR Addition

**Figure 2:**
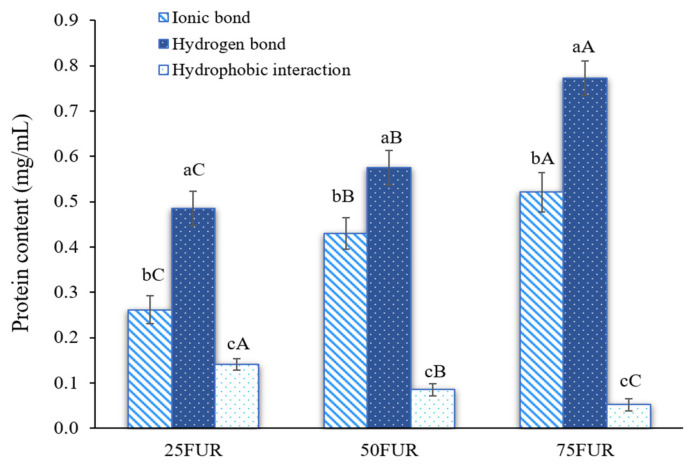
Intermolecular interaction forces of fish gelatin (FG) as affected by the addition of furcellaran (FUR) at different levels. Bars represent the standard deviation (*n* = 3). Lowercase letters on the bars indicate significant differences (*p* < 0.05) among different types of interaction within the same FUR level (intragroup comparison), while uppercase letters indicate significant differences (*p* < 0.05) of the same interaction type across different FUR levels (intergroup comparison).

Protein solubility from the fractional dissolution method has been used to determine the intermolecular forces of FG gels as affected by FUR addition (Figure 2). Stronger intermolecular forces formed in the protein-polysaccharide mixed gel correlated with greater protein solubility [23,41]. According to the findings, mixed gel samples had the ionic bond and hydrogen bond as major forces responsible for the mixed gel-forming system, with a subsequent synergistic effect arising from hydrophobic interaction. The addition of FUR resulted in a dose-dependent enhancement of both ionic and hydrogen bonding interactions (*p* < 0.05). Generally, hydrocolloid concentration is a predominant factor affecting the interaction between FG and anionic polysaccharides in water-based solutions [33]. FUR carries sulfate groups that are negatively charged, which could interact with the positively charged amino groups in the FG. A high concentration of FUR possesses a higher anionic group accessible for intermolecular interactions, leading to enhanced ionic bonding. During gelation, intramolecular or intermolecular hydrogen bonds were also formed between FG molecules (hydroxyl groups, carboxyl groups, amine groups, and some counterions) and FUR molecules (sulfate groups, hydroxyl groups, and other elements), which was evidenced by the increase in protein solubility [42]. Similarly, Wang, Han, Yu, Zhao, Pan, Prakash and Dong [41] studied interaction force in κ-carrageenan-gelatin hydrogels. They found that increasing the concentration of κ-carrageenan led to an increase in both ionic and hydrogen bonding, while hydrophobic interactions decreased. These findings also highlighted the influence of polysaccharide concentration on modulating different types of intermolecular forces within protein-based gel systems.

The interaction between FG and FUR influences the strength of the gel and the overall characteristics of the composite gels. Increasing the quantity of ionic and hydrogen bonds in the mixed system could provide stronger gel strength, as shown in Figure 1. However, the incorporation of a high level of FUR (>50%) resulted in an excess number of interactions, and consequently diminished gel strength for the blended gel. The previous study suggested that excessive cross-linking could lead to a rigid network that might hinder the flexibility of the gel and its strength [32]. This might refer to over-rigidity (less springiness) in the gel system, which affects a more fragile and easy-to-break gel under texture profile analysis. The decrease in springiness of the resulting gel when a high amount of FUR (>50%) was added has also confirmed this explanation (Table 1). Additionally, increasing the level of FUR in the mixed gel decreased the hydrophobic interaction. This might be associated with less attraction during the condensation of the FG and FUR processes at lower temperatures, which exhibited hydrophobic interaction forces [43]. Similar findings were reported in gelatin systems modified with high concentrations of carrageenan, which led to decreased hydrophobic interactions [41].

### 2.3. Changes in the Appearance of FG Gels as Influenced by FUR Addition

The contribution of FUR addition on the appearance of FG gels, defined as L*, a*, b*, and ∆E*, is presented in Table 2. The L* (lightness) of FG/FUR blended gels declined when the content of FUR incorporated rose (*p* < 0.05). Compared to other samples, the control sample (FG) exhibited the highest L* regarding all gel samples, while the gel containing 100FUR manifested the lowest L* (54.81) (*p* < 0.05). Petcharat, Benjakul and Hemar [27], Chilvers and Morris [44] suggested that coacervation between FG and FUR was proposed to reduce the L* in gelatin-mixed gels.

**Figure 3:**
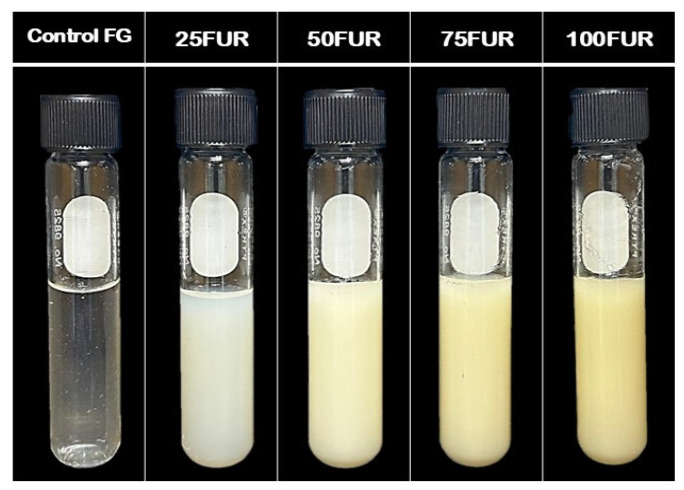
The photographs of fish gelatin (FG) as affected by the addition of furcellaran (FUR) at different levels.

Incorporating FUR to FG resulted in a reduction in L*, particularly at higher concentrations, as also evidenced by the formation of dull or opaque gels observed in Figure 3. Along with that, the incorporation of FUR enhanced the a* (redness) of the fish gelatin gel, with the control FG (without FUR added) showing the lowest a* (−2.20) among all the gels tested (*p* < 0.05). No statistically significant difference was detected between the FG/FUR mixed gel and the 100FUR gel (*p* > 0.05). In contrast, the b* (yellowness) of the sample increased as the FUR content increased in a dose-dependent manner (*p* < 0.05). The noticeable rise in the yellowness of mixed gel might be due to the natural coloration of FUR, which is white to pale yellow [45]. This result was similar to edible coatings that contained FUR; a lightly yellow-colored transparent appearance occurred when FUR was incorporated [46,47]. It was noted that the declines in L* and the rises in b* were in agreement with the change in the ∆E* (total difference in color) of FG/FUR blended gels when a higher amount of FUR was added. Control FG gel showed the lowest ∆E* with the highest L* (*p* < 0.05). The ∆E* of the FG/FUR blended gel exhibited a significant increase with the increase of FUR level. These results also showed a significant overall color change due to the increasing FUR concentration.

The visual comparison in Figure 3 reconfirmed that the color difference between the control FG and FUR-added samples was significant. As a consequence, furcellaran incorporation influenced the coloration of the fish gelatin blended gel via the ways that the hydrocolloids were disseminated and interacted within the gel matrix, as well as the inherent color of the hydrocolloid itself.

### 2.4. Syneresis of FG Gels as Influenced by FUR Addition

Syneresis refers to the expulsion of liquid from a gel as it contracts. Syneresis happens when the gel network is incapable of maintaining its structure. This phenomenon is observed in various types of gels, including gelatin gels [48,49,50]. Syneresis of all gel samples is presented in Table 3. When FUR was mixed, the elevations in the syneresis of gel samples increased (*p* < 0.05). In contrast, no significant differences were observed between 75FUR and 100FUR (*p* > 0.05). Among all hydrogels, the highest syneresis was observed in the 100FUR (0.44%), while the lowest syneresis was found in the control FG gel (0.13%) (*p* < 0.05).

Less gel syneresis has been pointed out as a positive characteristic of gelatin [36]. It was marked that there were no differences in syneresis between the 25FUR (FG containing 25% FUR) gel and the control FG (*p* > 0.05). As a result, the observed increase in syneresis of mixed gel samples might be attributed to the interaction between FG and FUR, resulting in a more compact structure, leading to decreased space for water entrapment. Previous studies also found that incorporating high levels of hydrocolloids such as agar, gellan, and κ-carrageenan with gelatin mostly leads to increased syneresis of the resulting gel [17,19,26]. Moreover, Petcharat, Chaijan and Karnjanapratum [30] stated that the incorporation of a low concentration of FUR as a solution form into sardine surimi (fish myofibrillar protein) could promote the development of a stronger network, which would enable the gel network to hold a greater amount of water. Based on the present study, adding FUR at a low level did not negatively impact the syneresis of the FG mixed gel.

### 2.5. Rheological Behavior Analysis of FG Gels as Influenced by FUR Addition

Besides gelatin bloom strength, rheology characteristics such as gelling and melting points of gelatin are key functional characteristics that influence the physical characteristics in real-world food applications [26]. The gelling and melting temperatures of FG gels, which are influenced by the different levels of FUR addition, are presented in Table 3. Control FG had the lowest gelling temperature among all samples (19.72 °C) (*p* < 0.05). The previous study generally reported a gelling temperature range of 8–25 °C for fish gelatin [51,52]. Herein, the gelling temperatures of the gel containing FUR continued to increase when the FUR concentrations were raised. It was highlighted that FG, in the absence of FUR, was unable to form a gel at an ambient temperature (>25 °C), meanwhile, all blended hydrogels containing FUR successfully achieved gelation at room temperature. The findings revealed that the ionic interaction between biopolymers (gelatin and hydrocolloid) might promote the gel-forming of the FG/FUR mixture. This was also reconfirmed that incorporating FUR led to an increase in ionic and hydrogen bonds, as shown in Figure 2. A related behavior was documented by Sinthusamran, Benjakul, Swedlund and Hemar [19] who stated that the gelling point of fish gelatin modified with κ-carrageenan was significantly affected by the percentage of hydrocolloid in the mixture.

All the FG/FUR samples exhibited significantly elevated melting temperatures in comparison to the control FG gel (*p* < 0.05). The rising melting point of blended gel samples corresponded with the elevated gelling point of FG/FUR mixtures. Higher gelling and melting temperatures were involved in the increasing number of chemical interactions required to generate a 3D gel network, as illustrated in Figure 2. As per the results, FG/FUR mixed gel is stable at room temperature (melting temperature > 25 °C), however, the addition of FUR (hydrocolloid) could maintain the thermoreversible characteristic of gelatin gel. Conversely, some hydrocolloids, such as gellan, might form an irreversible gel when incorporated with fish gelatin, in which the resulting gel could not melt within the temperature range studied (5–90 °C) [27]. The findings indicated that FUR significantly influenced the rheological properties of the composite gel. The presence of FUR greatly enhanced the thermostability of the FG/FUR blended gel, highlighted by the elevated gelling and melting points.

**Figure 4:**
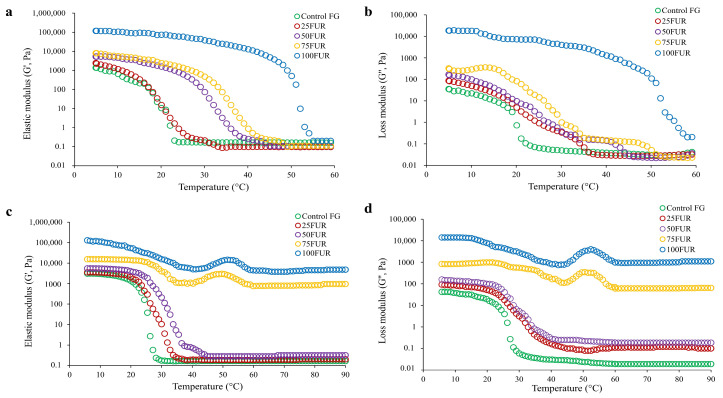
Elastic modulus (G′) (**a**) and loss modulus (G″) (**b**) of 6.67% fish gelatin (FG) as affected by the addition of furcellaran (FUR) at different levels during cooling from 60 to 5 °C. Elastic modulus (G′) (**c**) and loss modulus (G″) (**d**) of 6.67% FG as affected by the addition of FUR at different levels during heating from 5 to 90 °C.

Moreover, the changes in the rheological behavior of the FG/FUR mixed solutions were illustrated by elastic modulus (G′) and loss modulus (G″) upon lowering the temperature from 60 to 5 °C (Figure 4a,b) and raising the temperature from 5 to 90 °C (Figure 4c,d). The trends of G′ and G″ in all FG/FUR mixed solution samples throughout gelation and melting were generally the same. Both G′ and G″ of the FG/FUR mixed gel were higher than the control FG. The increase in FUR level caused the increase in G′ and G″ of the FG/FUR mixed gel. An increase in the elastic modulus implied that the gel network structure has been improved [40,53]. The findings suggested that incorporating furcellaran could markedly enhance the elastic modulus and loss modulus, thus increasing the melting point of the mixed gels.

### 2.6. Structural Property of FG Gels as Influenced by FUR Addition

#### 2.6.1. SAXS Profiles

**Figure 5:**
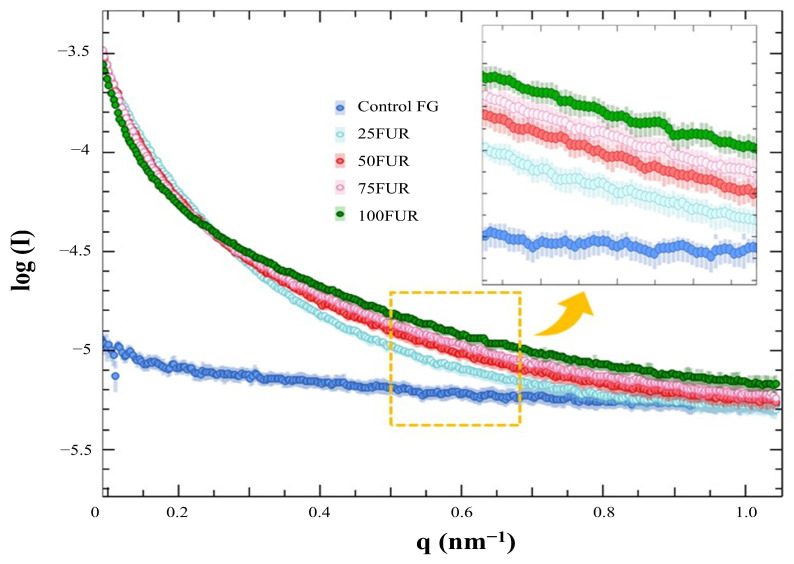
SAXS profiles fish gelatin (FG) gel as affected by the addition of furcellaran (FUR) at different levels.

The structural property of FG gels as influenced by FUR incorporation was evaluated by small-angle X-ray scattering (SAXS). The SAXS profiles were conducted at 4 °C and processed by using the SAXSIT4.41 software as presented in Figure 5. The observed SAXS profiles displayed distinct differences, with the control FG exhibiting the lowest scattering intensity value.

**Figure 6:**
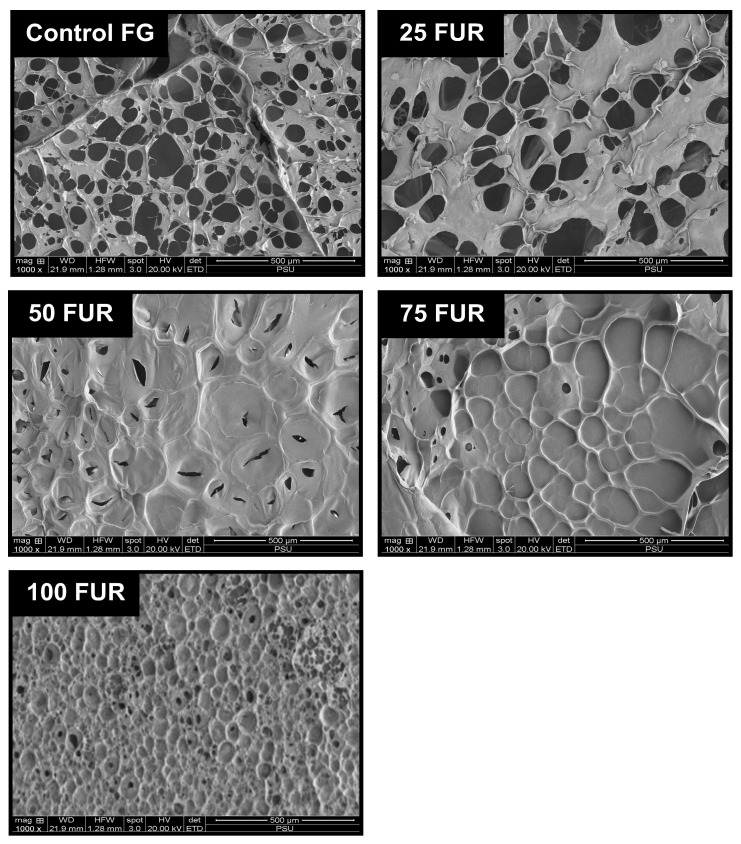
Microstructures of fish gelatin (FG) gel as affected by the addition of furcellaran (FUR) at different levels. Magnification: 1000×.

The SAXS scattering intensities of FG/FUR blended gels were raised with higher FUR percentages. This suggested that the incorporation of FUR caused a variation in electron density within the gel clusters and the matrix. A high number of intermolecular forces of FG/FUR mixed gel, particularly ionic interaction (Figure 2), might contribute to the increase in electron density. Similarly, Wisotzki, et al. [54] reported that the augmentation of covalent crosslinks through electron irradiation of gelatin hydrogel markedly enhanced the relative scattering intensity. Moreover, Ji, et al. [55] studied the morphology of gelatin blended with hydroxypropyl methylcellulose by using SAXS. They found that blended gels with higher hydroxypropyl methylcellulose contents could form denser networks, and the correlation length of the blended gels declines from 5.16 to 1.89 nm when the hydroxypropyl methylcellulose content augments from 1 to 6% [55]. In addition, the result was coincident with the gel microstructure observed via SEM (Figure 6); a denser gel structure with a smaller void was observed in a gel containing higher FUR levels. Therefore, the addition of FUR directly affects the structural properties of FG gel, leading to the formation of denser networks in mixed gels with higher levels of FUR.

#### 2.6.2. Microscopic Morphology

The microstructures of gel samples were visualized through SEM (Figure 6). Among all samples, control FG (without FUR addition) exhibited a coarser network with broader voids or holes. When the level of FUR increases from 25 to 75% FUR (25FUR, 50FUR, and 75FUR), the mixed gel structure shows larger strands with smaller voids. The biopolymer chains of FUR are integrated into the three-dimensional structure of FG, culminating in an enlarged network gel structure. FUR might also act as a filter to fill in the 3D network structure of the FG, resulting in a denser network structure in the mixed gels [18,30].

Moreover, the densest structure of the gel sample was observed in a gel containing pure furcellaran (100FUR). The interconnectedness of mixed gel samples was more noticeable after adding FUR. This was correlated with the greater intramolecular force, gelling, and melting temperature of the blended gel reinforced with a higher percentage of FUR (Figure 2 and Table 3). A corresponding incident was documented by Cheng, Zhang, Qiao, Yan, Zhao, Jia, Niu and Xu [18], Liu, Song, Wang, Wang, Ma, Zhao, Chen, Wang, Zhang and Wen [40]. The microstructure of gelatin-based composite gels was governed by κ-carrageenan added, following the positive change in rheological, physicochemical, and textural properties of composite gels. The present result reconfirmed that furcellaran could reinforce the fish gelatin network. Thus, the levels of FUR strongly impacted the gel network, which in turn influenced the physicochemical properties and viscoelastic characteristics of the mixed gel.

## 3. Conclusions

FG has become an important alternative source of gelatins. However, it has limited applications due to poor gelling properties, low gel strength, and gelling temperature. To alleviate such a drawback, the incorporation of FUR as a gelling agent significantly improved the properties of FG gels, especially when used at a level of 25% substitution. Enhanced gel strength, hardness, and reduced syneresis were achieved while preserving acceptable springiness. These improvements are attributed to strengthened intermolecular interactions, particularly increased ionic and hydrogen bonding between the biopolymers. Additionally, the changes in gel appearance and rheological properties highlight the impact of FUR on the gelling characteristics, especially since all FG/FUR mixed gels could be formed at room temperature. Microstructural analyses further confirmed that adding FUR resulted in a denser gel network. These findings suggest that FUR could be a promising additive for enhancing the functionality of FG, making it suitable for broader applications in the food and pharmaceutical industries. In addition, exploring the behavior of these gels under various storage and processing conditions, as well as their functionality in real food systems, will provide a more comprehensive understanding of their practical potential.

## 4. Materials and Methods

### 4.1. Materials

Furcellaran Estgel 1000 (food grade) was acquired from Est-Agar AS, located in Karla village, Estonia. It had a molecular weight of 2.55 × 10 ^5^ Da, and 9.7% moisture was used without additional purification. At 2.5%, furcellaran had 480 g bloom strength. Fish gelatin originated from tilapia skin and exhibited ~240 g bloom strength and was obtained from Lapi Gelatine S.p.A. (Lapi Gelatine S.p.A., Empoli, Italy).

### 4.2. Preparation of Fish Gelatin/Furcellaran Samples

Furcellaran (FUR) and fish gelatin (FG) powdered forms were initially dispersed and heated separately in DI water at 90 °C and 60 °C, respectively. The FG and FUR solution was mixed to obtain several FG:FUR ratios, namely 100:0 (Control FG), 75:25 (25FUR), 50:50 (50FUR), 25:75 (75FUR), and 0:100 (100FUR), *w*/*w*, in which the final concentration of the mixed solution equal to 6.67% (*w*/*v*). The solutions were stirred for 10 min until they achieved an equilibrium of homogeneity. The sample solutions were kept at 60 °C before they were utilized for gel formation. The pH of the samples was subsequently measured and recorded via a pH meter.

The obtained solutions were poured into cylindrical molds measuring 3 cm in diameter and 2.5 cm in height for setting gel. Before the analyses, all samples were stored at 4 °C for 18 h.

### 4.3. Analyses

#### 4.3.1. Evaluation of Textural Properties

- Gel strength

A texture analyzer (Stable Micro System, Surrey, UK) equipped with a 5 kg load cell and a cross-head speed of 1 mm/s was set to measure the gel strength of all samples at 8–10 °C, according to the method outlined by Xiong, Zhao, Zhang, Zhu, Liu, Zhang, Chen, and Fan [31]. A 1.27 cm diameter flat-faced cylindrical Teflon^®^ plunger was used. The maximum force (g) was recorded as the force reached when the plunger had penetrated 4 mm into the gel samples.

- Texture Profile Analysis

The gel samples were positioned on the TA-XT plus texture analyzer (Stable Micro System, Surrey, UK) with a 50 kg load cell, using a P/50 probe. The testing was conducted following the conditions of Sinthusamran, Benjakul, and Hemar [17]. The gel samples were positioned on the instrument’s base, and the tests were run with two compression cycles. TPA textural parameters were measured at 8–10 °C with the following testing conditions: crosshead speed of 0.5 mm/s, 50% compression of the original sample height. The time interval between the first and second compression was 10 s. The TPA parameters were derived from the force-time curves [24].

#### 4.3.2. Determination of Intermolecular Forces

The method recommended by Wang, Han, Yu, Zhao, Pan, Prakash and Dong [41] was used with slight modifications to examine the intermolecular forces of all samples. Gel sample 1 g was homogenized with 10 mL of SSA (0.05 mol/L NaCl), SSB (0.6 mol/L NaCl), SSC (0.6 mol/L NaCl + 1.5 mol/L urea), and SSD (0.6 mol/L NaCl + 8 mol/L urea). Thereafter, the samples were centrifuged at 1600 rpm for 4 min at 4 °C using an Eppendorf 5920R temperature-controlled centrifuge (Eppendorf North America Inc., New York, UK) to collect the supernatant [41,56]. The concentration of protein was quantified by the Folin-reagent method and labeled as SSA, SSB, SSC, and SSD. The differences between SSB and SSA, SSC and SSB, and SSD and SSC were used to determine the ionic bond, the hydrogen bond, and the hydrophobic interaction force.

#### 4.3.3. Measurement of Color

The color determination of hydrogels was conducted with an Ultrascan XE, Hunter Lab colorimeter (Hunter Lab Inc., Reston, VA, USA). The L*, a*, and b* were stated, and the total difference in color (∆E*) was calculated following the method of Jamróz, Kulawik, Krzyściak, Talaga-Ćwiertnia and Juszczak [29].

#### 4.3.4. Determination of Syneresis

The syneresis of hydrogels was evaluated as stated by Banerjee and Bhattacharya [57]. The mass of graduated centrifuge tubes without a sample (M_1_), the mass of gels together with the tubes after centrifugation (M_2_), and the initial mass of the sample (M_3_) were recorded. Then, the syneresis of the hydrogel was calculated using the formula (M_1_ − M_2_)/M_3_ and assigned as a percentage.

#### 4.3.5. Rheological Measurements

The MCR 302 rheometer (Anton Paar GmbH, Graz, Austria) was used to study the rheological properties of gels, following the method described by Sinthusamran, Benjakul, Swedlund and Hemar [19]. The measurement geometries included a parallel plate with probe PP25/P2 (25 mm), and the measuring gap was adjusted to 1.0 mm. The hot solution was loaded on the rheometer, and then temperature sweep mode cooling (60 to 5 °C) followed by heating (5 to 90 °C) was conducted. During oscillating, the conditions were fixed at a frequency of 1 Hz, a scan rate of 1 °C/min, and a strain of 1%. The values of the elastic modulus (G′) and the loss modulus (G″) were measured. Ultimately, the gelling and melting points were identified as the temperatures at which the ratio of G″ to G′, known as tan δ, equaled 1 (or δ = 45°).

#### 4.3.6. Small-Angle X-Ray Scattering (SAXS)

In situ small-angle X-ray scattering (SAXS) experiments were evaluated at beamline 1.3 W Synchrotron Light Research Institute (SLRI) (Suranaree University of Technology, Nakhon Ratchasima, Thailand). A monochromatic soft X-ray beam of 8 keV (ΔE/E = 0.93%) was used throughout the measurements. A small piece of each specimen was obtained directly from the center of the gel and sealed in a removable cell between two Kapton^®^ windows. The tests were conducted at 4 °C and repeated three times. The q-range for SAXS analysis was 0.04 to 0.7 nm⁻^1^. The specimen-to-detector distance for SAXS analysis was 5.18 m. The scattering data was analyzed with the SAXSIT4.41 software.

#### 4.3.7. Microstructure

The microstructures of hydrogels were evaluated via scanning electron microscopy (Quanta 400; FEI, Eindhoven, The Netherlands) in accordance with the procedure suggested by Liu, Song, Wang, Wang, Ma, Zhao, Chen, Wang, Zhang and Wen [40]. The hydrogels were rapidly quenched with liquid nitrogen, then cut into cubes about 3 mm, placed in an ultra-low temperature refrigerator at −80 °C for 2 h, and then freeze-dried to obtain the SEM samples. Dried specimens were affixed to a bronze stub, covered with gold, and subsequently evaluated at an acceleration voltage of 20 kV.

### 4.4. Statistical Analysis

Experiments were performed in triplicate. The data underwent analysis of variance (ANOVA). The means difference was determined using Duncan’s multiple range test (DMRT) [58]. All statistical analyses were conducted with SPSS Statistics Software Version 28 (SPSS Inc., Chicago, IL, USA). The *p* < 0.05 data was accepted as statistically significant.

## Figures and Tables

**Table 1:** Texture profile analysis (TPA) of fish gelatin (FG) gel as affected by the addition of furcellaran (FUR) at different levels.

Samples	Hardness (N)	Springiness	Cohesiveness	Gumminess (N)	Chewiness (N × mm)
Control FG	14.40 ± 0.28 ^b^	0.96 ± 0.01 ^a^	0.90 ± 0.03 ^a^	12.48 ± 0.18 ^b^	12.00 ± 0.23 ^b^
25FUR	18.42 ± 0.42 ^a^	0.95 ± 0.00 ^a^	0.88 ± 0.01 ^a^	14.69 ± 0.39 ^a^	14.02 ± 0.33 ^a^
50FUR	12.08 ± 0.23 ^c^	0.91 ± 0.01 ^b^	0.76 ± 0.02 ^b^	10.25 ± 0.46 ^c^	9.35 ± 0.41 ^c^
75FUR	9.34 ± 0.27 ^d^	0.79 ± 0.02 ^c^	0.66 ± 0.01 ^c^	8.33 ± 0.38 ^d^	6.61 ± 0.30 ^d^
100FUR	6.57 ± 1.26 ^e^	0.52 ± 0.07 ^d^	0.49 ± 0.04 ^d^	8.22 ± 0.31 ^d^	4.29 ± 0.52 ^e^

Different lowercase superscript letters in the column indicate significant differences (*p* < 0.05). Values are presented as mean ± SD (*n* = 10).

**Table 2:** Color values of fish gelatin (FG) gel as affected by the addition of furcellaran (FUR) at different levels.

Samples	L*	a*	b*	∆E*
Control FG	70.68 ± 0.50 ^a^	−2.20 ± 0.33 ^b^	8.85 ± 0.08 ^e^	21.18 ± 0.14 ^e^
25FUR	67.13 ± 0.55 ^b^	1.75 ± 0.03 ^a^	10.91 ± 0.05 ^d^	33.06 ± 0.12 ^d^
50FUR	63.34 ± 0.84 ^c^	2.00 ± 0.42 ^a^	13.28 ± 0.53 ^c^	34.75 ± 0.75 ^c^
75FUR	61.26 ± 0.29 ^d^	2.19 ± 0.20 ^a^	14.79 ± 0.68 ^b^	38.04 ± 0.54 ^b^
100FUR	54.81 ± 0.13 ^e^	2.15 ± 0.02 ^a^	16.14 ± 0.32 ^a^	41.27 ± 0.50 ^a^

Different lowercase superscript letters in the column indicate significant differences (*p* < 0.05). Values are presented as mean ± SD (*n* = 3).

**Table 3:** Syneresis, gelling, and melting temperatures of fish gelatin (FG) gel as affected by the addition of furcellaran (FUR) at different levels.

Samples	Syneresis (%)	Gelling Temperature (°C)	Melting Temperature (°C)
Control FG	0.13 ± 0.04 ^c^	19.72 ± 0.59 ^e^	26.38 ± 0.71 ^e^
25FUR	0.18 ± 0.03 ^c^	34.96 ± 0.43 ^d^	39.78 ± 0.57 ^d^
50FUR	0.26 ± 0.01 ^bc^	39.19 ± 0.17 ^c^	43.75 ± 0.39 ^c^
75FUR	0.39 ± 0.15 ^ab^	41.75 ± 0.13 ^b^	49.64 ± 0.11 ^b^
100FUR	0.44 ± 0.10 ^a^	50.76 ± 0.30 ^a^	69.18 ± 0.66 ^a^

Different lowercase superscript letters in the column indicate significant differences (*p* < 0.05). Values are presented as mean ± SD (*n* = 3).

## Data Availability

The data presented in this study are available in this article. Further inquiries can be directed to the corresponding author.
